# Thromboembolic Complications of SARS-CoV-2 and Metabolic Derangements: Suggestions from Clinical Practice Evidence to Causative Agents

**DOI:** 10.3390/metabo11060341

**Published:** 2021-05-25

**Authors:** Francesco Nappi, Adelaide Iervolino, Sanjeet Singh Avtaar Singh

**Affiliations:** 1 Centre Cardiologique du Nord de Saint-Denis, Department of Cardiac Surgery, 93200 Saint-Denis, France; 2 Department of Cardiovascular Sciences, Fondazione Policlinico Universitario A. Gemelli IRCSS, 00168 Rome, Italy; adelaide.iervolino@libero.it; 3 Department of Cardiothoracic Surgery, Golden Jubilee National Hospital, Agamemnon St, Clydebank G81 4DY, UK; Sanjeet.Singh@gjnh.scot.nhs.uk

**Keywords:** thrombosis, SARS-CoV-2 infection, cytokines, metabolism, inflammation

## Abstract

Severe Acute Respiratory Syndrome (SARS) Coronavirus (CoV)-2 is a recently identified positive sense single-strand RNA (ssRNA) β-coronavirus. The viral spike proteins infect human hosts by binding to the cellular receptor angiotensin-converting enzyme 2 (ACE2). The infection causes a systemic illness involving cell metabolism. This widespread involvement is implicated in the pathophysiology of the illness which ranges from mild to severe, requiring multi organ support, ranging from oxygen supplementation to full cardiovascular and respiratory support. Patients with multiple co-existing comorbidities are also at a higher risk. The aim of this review is to explore the exact mechanisms by which COVID-19 affects patients systemically with a primary focus on the bleeding and thrombotic complications linked with the disease. Issues surrounding the thrombotic complications following administration of the ChAdOx1 nCoV-19 (Astra-Zeneca-Oxford) vaccine have also been illustrated. Risk stratification and treatment options in these patients should be tailored according to clinical severity with input from a multidisciplinary team.

## 1. SARS-CoV-2 Pathogenesis, Hemostatic Alterations and Metabolism Interference of Therapeutical Agents

### 1.1. SARS-CoV-2 Infection Strategy and Host Immune Response

Severe Acute Respiratory Syndrome (SARS) Coronavirus (CoV)-2 is a recently identified positive sense single-strand RNA (ssRNA) β-coronavirus. The viral spike proteins infect human hosts by binding to the cellular receptor angiotensin-converting enzyme 2 (ACE2) [1]. It is highly expressed on alveolar epithelial type 2 (AT2) cells, tubular epithelium of the kidney, cardiac myocytes, enterocytes, endothelial cells and other human tissues as well [2,3].

The consecutio temporum between transmission of SARS-CoV-2 infection and involvement of pulmonary, cardiac, endothelial, and blood cell metabolism assumes important relevance in preventing the devastating effects of the Covid-19 disease. After its transmission, which occurs primarily from inhalation of viral particles, the second stage involves localization in the cells of the respiratory tract [2]. Since the virus can survive for 24–72 h on surfaces, with differences depending on the type of surface and where it is located, viral transmission can be greatly amplified [4]. Infected people experience symptoms comparable to many viral infections with the onset of systemic spreading of COVID-19 leading to fever, fatigue, headache, cough, shortness of breath, diarrhea, and myalgia [5,6,7]. Covid-19 has the potential to cause severe damage to many tissues, including systemic inflammatory response syndrome (SIRS), acute respiratory disease syndrome (ARDS), multiorgan involvement, and shock [8]. One of the most feared complications is thromboembolism development which leads to severe clinical phenotypes: worsening of pulmonary conditions, oxygen desaturation, and acute respiratory distress. Older patients with multiple comorbidities, predominantly of cardiovascular interest, have a higher risk of developing severe disease compared to younger patients who, even if in good health conditions, are at risk of complications [9]. This trend has now been reversed by the presence of genetic variants due to the viral mutation which has increased its contagion capacity [10,11,12].

### 1.2. Alteration of Hematological Parameters from Clinical Experience of Thromboembolic Events

Laboratory tests performed on patients with Covid-19 have shown lymphopenia [5], mild thrombocytopenia and increased lactate dehydrogenase. In addition, the markers of inflammation such as C-reactive protein, D-dimer, ferritin, and interleukin-6 (IL-6) were altered [13]. High levels of IL-6 have been linked with disease severity and a procoagulant profile with an evolution towards more severe complications in frail patients [14].

Among patients with Covid-19 who required hospitalization due to their critical condtion, the most common haemostatic anomalies were mild thrombocytopenia [15] and increased levels of D-dimer [16]. Cui et al. noted that Covid-19 patients presenting with venous thromboembolism (VTE) had higher D-dimer levels compared to non-VTE patients. A large series of patients studied in the first phase of the SARS Cov 2 epidemic in China, who were hospitalized in severe clinical condition, showed higher D-dimer levels than those in whom the disease was less severe [2,6,13,17,18,19]. The evidence that D-dimer was a negative prognostic factor in non-survivors compared to survivors also emerged from other studies assessing patients hospitalized in intensive care units. Zhang L et al. [20] used a cutoff value of D-dimer of 2.0 μg/mL and reported a mortality rate of 0.37% for patients with values <2.0 μg/mL (1 of 267) compared to 17.9% for patients with values ≥2.0 μg/mL (12 of 67). However, using the same cutoff for D-dimer, there were differences in mortality rate with a lower range between survivors (10.4%) and non-survivors (18%) (<2.0 μg/mL; 8 out of 77 patients vs. ≥2.0 μg; 17 of 93) suggesting a potential selection bias [21].

Changes in blood coagulation status led to an increased risk of receiving mechanical ventilation, requiring hospitalization in an intensive care unit (ICU), or incurring death. A combination of a paucity of data alongside unclear and conflicting data supports the validity of other blood coagulation tests [22,23]. Evidence has shown that the variability in disease severity is due to a prolongation of prothrombin time (PT), a difficulty in normalizing the international normalized ratio (INR) [2,13,24] and the thrombin time (TT) [25]. Patients who remain in critical condition tend to maintain a reduced activated partial thromboplastin time (aPTT) [2,6,9] (Table 1).

Evidence suggesting a role for platelets in viral infections has long been proven. For example, the presence of influenza virus type A (IAV) particles was noted in the platelets of patients with acute influenza infection. Once the IAV was incorporated into the platelets, TLR7-dependent C3 was released with the subsequent activation of neutrophils and neutrophil extracellular traps (NETs) release [34]. Platelets play an essential role in maintaining vascular integrity but can trigger thrombogenic mechanisms. More recently, the platelets role in viral infections has been studied highlighting its active participation in the host’s immune response [35]. The enormous amount of data available on the pathophysiological mechanisms that support SARS-CoV-2 infection have clearly shown that during viral infections, the risk of thrombosis is higher. A recent review discussed the potential role of platelets in thrombosis from COVID-19 infection. It confirmed the Chinese study investigating the close correlation between thrombocytopenia and risk of in-hospital mortality [36,37].

### 1.3. Interference of Antiviral Drugs with Antiplatelet and Anticoagulant Medications

The optimal therapy to be recommended for patients with severe Covid-19 disease has been tested and several drugs have been used to prevent the progression of the disease. The interactions that some of these drugs have with antiplatelet and anticoagulant agents have been clinically documented and considered important. The administration of experimental drugs has often been associated with excessive risk, as well as a reduced risk, of thrombotic events or thrombocytopenia in previous studies in non-Covid-19 populations and more recently in patients with Covid-19.

Bevacizumab has been associated with the occurrence of cardiovascular adverse events including MI, stroke and VTE. Bevacizumab is a monoclonal antibody that interferes with vascular endothelial growth factor (VEGF) and is being investigated for COVID-19 [38,39]. Another substance being tested is fingolimod. It is an immunomodulating agent that has been tested in patients with Covid-19 and is able to reduce reperfusion damage after neurological complications from stroke by improving outcomes [40].

Hydroxychloroquine, which has been the subject of open debates supported by opposing points of view, has since obtained FDA (food and drug administration) clearance for the treatment of COVID-19; however, it can potentially exert the antithrombotic action through interference with antiphospholipid antibodies [41].

**Figure 1:**
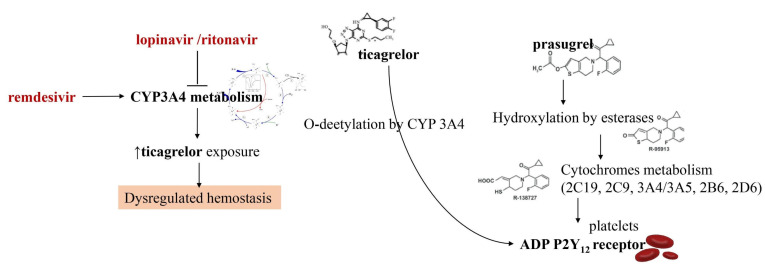
Interaction between antiviral agents and antiplatelet drugs on CYP3A4 metabolism. In red, antiviral agents are depicted. Lopinavir and ritonavir exert an inhibitory action on the cytochrome. This increases the exposure of ticagrelor leading to a dysregulation of hemostasis (highlighted in the picture being it the only depicted potential effect). Remdesivir is instead an inducer of CYP3A4 function. Differently from ticagrelor, prasugrel is metabolized by several cytochromes (2C19, 2C9, 3A4/3A5, 2B6, 2D6), thus its effects seem to be unmodified by ritonavir or lopinavir interaction. CYP3A4: Cytochrome P450 3A4, ADP P2Y12 receptor: adenosine 5′diphosphate P2Y12 receptor.

In this last year, researchers have directed a big commitment towards the study of drugs for the treatment of COVID-19 that may have interfered with oral antiplatelet drugs in oxidative processes at the level of hepatic cytochrome P450 (CYP) enzymes. The consequence is that the action of many antiviral drugs affects platelet metabolism. For example, the administration of lopinavir or ritonavir, protease inhibitors acting on CYP3A4 metabolism, may result in the inhibition of platelet activity at the level of CYP3A4 metabolism. As the active metabolite of clopidogrel is formed mainly by oxidation in CYP2C19, it leads to a reduction in the effective dose of clopidogrel. Instead, the oxidative process leading to the active ticagrelor metabolite occurs in CYP3A4 which is inhibited by lopinavir and ritonavir. The result is an increase in the effect of ticagrelor [42,43,44,45] (Figure 1).

The direct consequence of the combined use of these drugs is dysregulation of the haemostatic process which must be properly evaluated. Although the solution to this drawback is the use of P2Y12 platelet function tests to guide the use of clopidogrel or ticagrelor, there is limited clinical data available to support this strategy. A drug that is not affected by the action of lopinavir/ritonavir is prasugrel which may be a viable option in the absence of contraindications [42,43,44,45]. The effects of remdesivir appear to be different. This antiviral, which is a nucleotide analog inhibitor of RNA-dependent RNA polymerase, induces the function of CYP3A4. This may cause enzyme induction necessitating dose adjustments for oral antiplatelet drug agents that are currently not recommended. It is important to point out that significant drug interactions between different investigational therapies for Covid-19 and parenteral antiplatelet drugs such as cangrelor and glycoprotein IIb/IIIa inhibitors have not been reported.

We are aware of interactions between experimental drugs used for COVID-19 and therapies with oral anticoagulants administered for various cardiovascular diseases. Ritonavir use has also been shown to affect the choice and dosage of many anticoagulant agents. Careful surveillance after administration with vitamin K antagonists (VKAs), or apixaban and betrixaban, is required to make a dosage adjustment. The combination of drugs such as edoxaban and rivaroxaban and lopinavir/ritonavir administered contemporary is not recommended [46]. Although administration of tocilizumab which inhibits IL-6, increases the expression of CYP3A4; however, specific indications are lacking to adjust anticoagulant therapy when this anti-inflammatory is pharmacologically coupled in the treatment of Covid-19. To the best of our knowledge, we cannot affirm the evidence of major pharmacological interferences between the experimental COVID-19 therapies and the administration of anticoagulants for parenteral access.

## 2. Venous Thromboembolism Diagnosed in Covid-19 Patients and Management

### 2.1. Clinical Diagnoses of Venous Thromboembolism

We are aware of a small number of studies that have reported the incidence of VTE in patients with Covid-19 [47,48]. A Chinese retrospective study worked in this direction by reporting a percentage of 25% (20 out of 81) of patients admitted to the ICU in whom an accident of VTE occurred. Of note, none of the patients had been managed with the use of VTE prophylaxis drugs [49]. Klok et al. [50], in a multicenter study that included 184 patients with severe Covid-19, recorded the percentage of 31% (95% confidence interval: 20% to 41%) of patients who developed an accident of VTE. All patients had been treated with VTE prophylaxis drugs, although the authors noted underdosing in 2 of the 3 recruiting centers (81). It cannot be ruled out that VTE may go undiagnosed and unrecognized in patients with severe Covid-19. This is an important aspect during the clinical evolution of Covid-19, as ARDS in those patients is potentially the cause of a vicious circle involving hypoxia, pulmonary vasoconstriction, pulmonary hypertension and right ventricular failure. The onset of pulmonary embolism is an additional clinical event that often cannot be resolved.

### 2.2. Medical Treatment in the Acute Setting and on Discharge

The use of drugs for systemic anticoagulation has been a crucial point for the treatment of VTE. The choice of the most suitable drug requires clinical considerations factoring in comorbidities and possible impairment of renal or hepatic function, haematological disorders such as thrombocytopenia or that of the gastrointestinal system. Multiple therapies may be considered including a change in the anticoagulant pharmacological treatment in progress during the hospitalization period, due to the critical state at hand to a more suitable regiment at the time of discharge to adapt to the convalescence period.

This pharmacological management used in critically ill hospitalized patients with VTE, in whom the administration of parenteral anticoagulants such as, for example, UFH may be preferred because it has good pharmacodynamic and pharmacokinetic requirements without known drug interactions with experimental Covid-19 therapies. It is important to underline that the effects of the therapy are obtained after a longer period with the administration of UFH (unfractionated heparin) because they depend on the achievement of a therapeutic aPTT. In addition, healthcare workers are more exposed to the risk of contamination due to frequent blood draws. Therefore, these aspects argue for the preferential choice in the administration of LMWH in patients in whom invasive procedures are not planned.

The use of oral anticoagulation with DOAC is certainly advantageous because it does not involve the need for monitoring, easing the planning of discharge, and for the outpatient management of the patient. It is possible that the worsening of the patient’s clinical condition including, for example, the deterioration of the respiratory function in patients with Covid-19, may necessitate a switch between these classes of medications. Patients who are expected to be discharged are advised to use DOAC or LMWH to limit contact with healthcare personnel because controls for INR monitoring as for vitamin K antagonists (VKA) are not required.

### 2.3. Pulmonary Embolism in Covid-19 Patients: Stratification and Choice of Therapy

The vast majority of patients with Covid-19 and symptoms referable to acute DVT should be treated at home with anticoagulant therapy whenever possible. However, there are a number of patients for whom hospitalization is necessary to initiate acute endovascular techniques such as local fibrinolysis or embolectomy and patients with refractory symptoms [51]. Patients at medium and high risk for VTE may need the input of a multidisciplinary team to manage their PE [52,53,54,55]. The available data are limited but report a lower mortality with the use of advanced therapies for the treatment of VTE. The caveat here is the limited data which marginally demonstrates lower mortality from routine use of advanced VTE therapies. Therefore, severe respiratory failure sustained by a PE treated with the use of catheter-guided therapies during the pandemic should be limited to the most critical patients. Likewise, the indiscriminate use of filters positioned in the inferior vena cava should be avoided [56]. The placement of a filter is recommended in restricted cases for patients experiencing recurrent PE despite optimal anticoagulant treatment or with clinically significant VTE in the context of absolute contraindications to anticoagulant therapy [57]. IVC filters do not exonerate patients from the use of anticoagulation which must be restored immediately, with the dosage of the drug gradually increased whilst considering the potential to bleed. Patients with intermediate-risk and hemodynamically stable [52,58,59,60] should be closely monitored and managed with anticoagulant therapy.

Rescue systemic fibrinolysis should be considered in patients with progressive deterioration, either systemically or by transcatheter approach. Instead, for patients with evident haemodynamic instability and high-risk PE [52,58,59,60] systemic fibrinolysis is indicated with two possible options, a percutaneous catheter approach or if this is contraindicated, systemic fibrinolysis.

### 2.4. Management of Antithrombotic Drugs in Patients with SARS-CoV-2 Infection and Critical Illness

Patients with severe SARSCoV-2 infection and critical illness have a higher risk of VTE. Many factors contribute to the development of VTE which is often associated with worsening clinical condition and rapid deterioration of the patient. First, they have haemostatic imbalance coupled with a systemic inflammatory state and are forced into long immobility because they are assisted with mechanical ventilation. Second, the placement of central venous catheters contributes to increasing the risk of VTE in the ICU and infections [61,62,63]. Third, nutritional shortage and liver dysfunction can interfere with the production of clotting factors [64]. A modification of the pharmacokinetics is to be considered in critically ill patients who require dosage adjustment of anticoagulant drugs [65], caused by factors related to the absorption, metabolism, and renal or hepatic elimination of these drugs to be considered with the possible presence of organ dysfunction.

Administration of anticoagulant therapy by systemic parenteral infusion is recommended in majority of patients whereby Covid-19 with acute thrombosis occurs. In these cases, the use of UFH can be used based on expected protocols, or in patients with deterioration of renal function. In the absence of an application for an urgent procedure, the use of LMWH is a reasonable alternative [66]. Anticoagulation is of particular importance in patients undergoing ECMO (extracorporeal membrane oxygenation) for whom continuous monitoring of anticoagulant drugs is required to maintain the patency of the circuit especially when hemodynamic stability is ensured by lower blood flow.

Available data are scarce to establish complication rates in SARS-CoV-2 patients, but thrombosis and haemorrhage rates can be high reaching the rate of 53% and 16% respectively as reported in other populations with respiratory failure [67]. Even more limited are the data from the surveillance of ECMO patients in critical condition for SARSCoV-2 infection. Two studies reported results with very high mortality which was five out of six patients in one series and three out of three in another [13,24]. To date concerns related to recommend target anticoagulation therapy for critically patients with overt disease from severe SARS Cov 2 infection requiring ECMO exists due to lack of insufficient data [68].

### 2.5. Risk Stratification Scores to Drive Pharmacological Prophylactic Treatment

Patients with Covid-19 and a lung infection who required hospitalization following clinical complications have an increased risk of VTE [52,69]. The use of prophylactic anticoagulation and the dosage are recommended by current guidelines and the position papers of professional societies, predominantly based on large randomized clinical trials that have reported a benefit concerning outcomes after hospitalization [52,70,71,72].

Several studies have reported that early initiation of appropriate prophylactic anticoagulation reduces the risk of VTE in critically ill patients [73,74,75]. The Caprini score, the International Registry of Medical Prevention on Venous Thromboembolism (IMPROVE) model and the Padua model are risk stratification tools useful for VTE risk assessment [76,77,78,79,80,81]. For example, the use of the Padua model was reported by Wang et al. highlighting that 40% of patients admitted to hospital for Covid-19 and in critical condition were at high risk of VTE. However, the study lacks data on the use either of prophylaxis for VTE or about the incidence of VTE [82]. VTE drug prophylaxis is indicated for patients admitted to the hospital for COVID-19 who have respiratory failure or comorbidities such as active cancer and heart failure [83]. This therapy should also be extended to patients with prolonged bed rest and who require a prolonged admission to the intensive care unit unless patients have specific contraindications.

In January 2020 a WHO report recommended interim guidance for daily prophylaxis with low molecular weight heparin (LMWH) or unfractionated subcutaneous heparin (UFH) to be administered twice daily [84]. In patients forced into prolonged immobilization, in whom prophylactic pharmacological treatment is contraindicated, the use of mechanical prophylaxis with intermittent pneumatic compression of the VTE should be considered [84,85]. The administration of pharmacological prophylaxis for VTE should be very rigorous to avoid episodes of failed doses which has been a common occurrence leading to worse outcomes [86]. Pregnant patients with Covid-19 deserve particular consideration and must be meticulously evaluated. This population of individuals have a higher risk of VTE that also extends to the postpartum period [87,88]. To date, there are insufficient data to draw definitive conclusions on pregnant women who have been admitted to hospital with Covid-19 even if a greater risk of VTE is conceivable. VTE risk stratification for this patient population helps consider the use of pharmacological thromboprophylaxis, especially if they have other risk factors for VTE. Further research is needed to establish the appropriate prophylactic dosage of anticoagulants based on the weight of pregnant patients [89].

It is important to point out that the problem of extended prophylaxis with LMWH [90] or direct oral anticoagulants (DOAC) involves patients once discharged from the hospital after the resolution of the acute episode of Covid-19 [91,92,93,94]. In discharged patients, although the risk of VTE is reduced, the occurrence of bleeding events is increased [95,96]. There is no detailed information that clarifies this aspect of Covid patient management. However, it would be useful to employ individualized risk stratification models for thrombotic and haemorrhagic episodes when considering prolonged, 45-day prophylactic anticoagulant therapy. The latter is indicated in discharged patients with a high risk of VTE, with reduced mobility or with active neoplastic disease, who may have a high D-dimer > 2 times the upper limit, but who are at low risk of bleeding [93,97,98].

## 3. Hypotheses of Thrombosis Generation and Pathophysiological Mechanisms

### 3.1. Covid-19 and Disseminated Intravascular Coagulation

Patients with clinically critical Covid-19 have a greater chance of developing disseminated intravascular coagulation (DIC) [99,100] which is common to many diseases that evolve with severity [101]. It has not yet been demonstrated whether Covid-19 can lead to the development of a DIC with mechanisms intrinsic to SARS Cov 2 that cause direct activation of coagulation cascades. The hypercoagulable state caused by DIC leads to an activation of the tissue factor pathway with consequent platelet consumption and a consistent bleeding diathesis. It is characterized by raised fibrin degradation products (FDPs) which are commonly elevated in Covid-19 patients suffering from thrombosis.

The ISTH DIC score calculator is commonly used to establish the diagnosis of DIC [102]. The worsening of blood coagulation in patients with a critical picture of Covid-19 is carried out by monitoring platelet counts, PT, D-dimer, and fibrinogen. The continuous monitoring of the parameters listed above is the first step for correct identification of the DIC and starting its management. The appearance of bacterial superinfections is not uncommon and must be promptly treated with aggressive antibiotic therapy. The sudden prophylaxis with LMWH can decrease the formation of thrombin and reduce its consumption, to be useful in modulating the evolution of DIC. Preliminary results are based on limited data which, however, have shown an effective benefit with the prophylactic administration of LMWH [93,96]. Regarding the discontinuation of long-acting single or dual antiplatelet therapy (DAPT), it is recommended in most patients when the diagnosis of DIC is suspected, administration of antiplatelet medications in patients who have suffered from a recent ACS or who have implanted stents are left alone. For patients with a moderate or severe clinical condition of COVID-19 in whom dual antiplatelet therapy is indicated because they have received PCI in the past 3 months or have suffered from recent MI, even in the presence of a suspected or confirmed diagnosis of DIC but without noticeable bleeding, therapy must be individually tailored. In the absence of strong evidence confirming a DIC it is reasonable to continue the DAPT, if the patient has a platelet count of at least 50,000. The administration of a single antiplatelet drug is indicated when the platelet count is between 25,000 and 50,000 while an interruption of antiplatelet therapy is recommended for a platelet count of less than 25,000. However, rigidity in the application of these indications is not necessary and therapy must be adapted according to the patient’s condition, increasing or decreasing the antiplatelet dose and paying close attention to the possible onset of thrombotic complications or bleeding. Parenteral systemic fibrinolysis with the consequent elimination of thrombi scattered in the organs favors the resolution of DIC.

Patients with Covid-19 rarely suffer from clinically evident bleeding. However, significant blood loss is common in patients who develop DIC often when septic coagulopathy is associated [103]. If bleeding occurs, treatment consists of transfusion of blood products.

First, the use of platelet concentrate is useful in patients who have DIC with active bleeding to maintain platelet counts greater than 50 × 10^9^/L or in patients who have a high risk of bleeding because they require more invasive procedures to maintain a platelet count greater than 20 × 10^9^/L. Second, the administration of fresh frozen plasma (15 to 25 mL/kg) in patients who experience active bleeding and whose PT or aPTT values are altered (PTT> 1.5 times normal) or who have a decrease in fibrinogen (<1.5 g/L). Third, the administration of concentrated fibrinogen or cryoprecipitate for patients in whom the values of fibrinogen are low and persistent over time (<1.5 g/L) and the administration of prothrombin complex concentrate where transfusion of fresh frozen plasma is not possible. There are currently no indications for the routine use of tranexamic acid in patients with DIC associated with COVID-19 [104].

### 3.2. Direct Viral Damage and Endothelitis-Driven Inflammatory Reaction

Concerns about the triggering of changes in the hemostatic process may be also related to the direct effect of SARS-CoV-2 viral particles. Varga et al. demonstrated the presence of viral elements within endothelial cells due to the expression of ACE2 receptors. Also, inflammatory cells were demonstrated, corroborating evidence of a direct viral involvement towards endothelitis development, also noted in several organs [105].

Preclinical analyses have also demonstrated the successful infection of organoids by viruses confirming their tropism for endothelial cells. Investigators used clinical-grade soluble human ACE2 to achieve the infection [106,107]. Endothelial damage further contributes to inflammatory cell infiltrate in the lungs worsening the respiratory parameters [108].

### 3.3. Covid-19-Associated Hyperinflammatory Syndrome (cHIS)

A further hypothesis for thrombosis generation is related to cytokine storm precipitating an initially controlled inflammation in the more complicated SIRS [101], as emerged from the observation of similar cases of individuals infected with other viral diseases such as HIV, Zika, Chikungunya, and Ebola [109,110,111].

**Figure 2:**
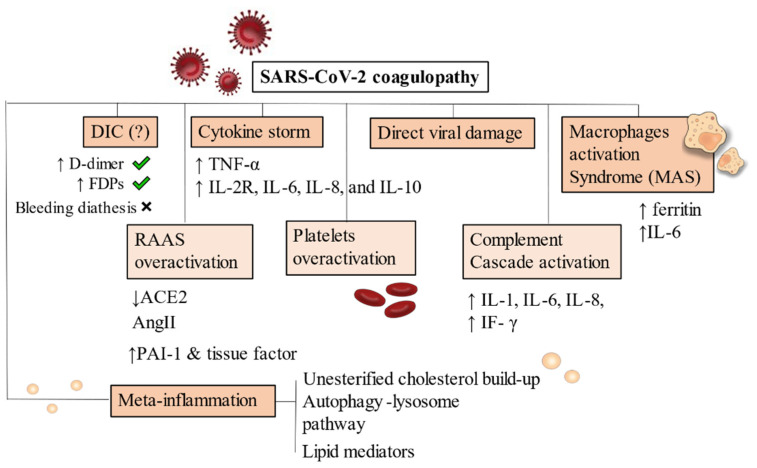
Pathophysiology of SARS-CoV-2 coagulopathy. DIC has been frequently noticed in Covid-19 severe patients but bleeding diathesis was a less present feature. Cytokine storm and inflammatory-driven thrombogenesis is the most known hypothesis, due to IL-2R, IL-6, IL-8, and IL-10 cascade generation. A direct viral damage has also been acknowledged to start endothelitis and endothelial damage. MAS is instead an added mechanism present on an already compromised immune condition where hyperferritinaemia and increase in IL-6 production are pathognomonic of macrophages overactivation. RAAS system, complement and platelets also drive uncontrollable responses by generating hypercytokinemia and dysregulating fibrinolysis. Increased levels of PAI-1 and tissue factor have been demonstrated. Meta-inflammation is another possible trigger for coagulopathy. Obesity, hyperinsulinemia and metabolic syndrome are strong risk factors for severity of infection in hospitalized patients. Primary conditions developing after viral infection are depicted with a darker background. RAAS overactivation, platelets and complement activation are mainly secondary mechanisms, thus they appear in a lighter background. *Abbreviations*: DIC: disseminated intravascular coagulation, FDPs: fibrin degradation products, ACE: angiotensin converting enzyme, AngII: angiotensin II, PAI-1: plasminogen activator inhibitor, IF-γ: interferon- γ, TNF-α: tumor necrosis factor-α, IL-6, IL-8, IL-10: interleukin 6, interleukin 8, interleukin 10. IL-2R: interleukin 2 receptor.

As a consequence of dysregulated immune reactions, hypercoagulable states are established due to continued inflammation. Macrophages activation syndrome (MAS) and cytokines released play major roles by activating a pro-inflammatory cascade [112,113] (Figure 2).

Recently, Webb et al. [114] proposed several criteria to diagnose the Covid-19-associated hyperinflammatory syndrome (cHIS) which has been associated with coagulopathy and hypercytokinemia leading, among the other complications, to mechanical ventilation and death in most severe cases. Criteria for the diagnosis have not been established at the time of writing. Thus, the authors proposed six of them: fever, hyperferritinaemia from macrophages activation syndrome, haematological dysfunction (N/L, neutrophil to lymphocyte ratio), hepatic injury (lactate dehydrogenase or aspartate aminotransferase), D-dimer to diagnose coagulopathy and cytokinaemia (C-reactive protein, interleukin-6, or triglycerides levels).

## 4. Inflammatory Cascade: The Bridge between Metabolic Derangements and Thrombogenesis

### The Immunologic Dialogue of Cytokines, T Cells, and Checkpoint Proteins Accelerating Thrombosis in Atherosclerotic Lesions

Endothelial dysfunction, which is the first recognizable step of Covid-19 thrombogenesis from an inflammatory cause, is first seen in atherosclerosis development. Metabolic derangements play main roles as atherogenic stimuli such as dyslipidemias, hypertension, and obesity which stimulate a series of changes in lesion-prone vascular endothelium, towards a vasoconstrictor, prothrombotic, proliferative, inflammatory phenotype. Pro-inflammatory cytokines, oxidized lipoproteins (ox-LDL), and advanced glycation end products (AGE), as well as disturbed blood flow associated with reciprocating, low shear stress [115] lead to endothelial activation (EA). These signals transduce mainly via the pleiotropic transcription factor TF nuclear factor-κB (NF-κB), resulting in a coordinated program of genetic regulation within the endothelial cell, among which chemokines, surface expression of adhesion molecules (e.g., vascular cell adhesion molecule-1 [VCAM-1), and prothrombotic agents such as tissue factor, von Willebrand Factor and plasminogen activator inhibitor (PAI-1) [116,117].

According to the Virmani classification, the intimal xanthoma is the initial detectable small lesion constituted by foam cells with intracellular- and *not extracellular*- lipid accumulation.

The endothelial expression of adhesion molecules VCAM-1 and ICAM-1 drives leukocytes and platelet recruitment. Also, smooth muscle cells coming from the tunica media, migrate to the plaque attracted by the PDGF stimulus and start to produce other inflammatory cytokines, such as IL-1 and IL-6.

An interesting role is one of the activated T cells, mainly CD4^+^: the recognition of proteic antigens presented by macrophages on MHC type II molecules triggers a response for which Th1 cells produce IFN-ɣ, in turn activating macrophages and overly stimulating the synthesis of pro-atherogenic inflammatory cytokines. In this process, costimulatory and coinhibitory receptors on T cells direct T-cell function and determine T-cell fate. These co-signaling molecules are divided into pro-atherogenic and anti-atherogenic ones. It is known, in fact, at least in the pre-clinical setting, that CTLA-4 blocking accelerates atherosclerosis development and decreases luminal patency [118,119].

The evolution of the plaque sequentially favours the formation of pathological intimal thickening (PIT) lesions consisting of macrophage foam cells and small extracellular lipid pools. The lesions eventually progress to fibrous cap atheroma (FCA) presenting with a fibro-adipous cap and necrotic cores from extracellular lipids, cholesterol crystals, and calcifications due to the accumulation of osteocalcin, osteopontin, and Bone Morphogenetic Protein (BMP). The last stages of atherogenesis involve mainly complicated lesions with fissuration and erosion resulting in thrombus which provokes a critical reduction of blood flow, thus occluding the vessel.

During plaque progression, regulators of immune cells divide into pro-atherogenic immune checkpoint proteins CD28–CD80/86, OX40–OX40L, CD137–CD137L and CD30–CD30L and anti-atherogenic ones CTLA-4–CD80/CD86, PD-1–PD-L1/2, ICOS–ICOSL, GITR–GITRL, CD27–CD70 and TIM proteins [120]. CTLA-4 is mainly expressed on Tregs and activated CD4^+^ and CD8^+^ T-cells but also on monocytes and activated B cells. Their anti-atherogenic role has been demonstrated by preclinical and clinical studies, without a clear elucidation of the mechanism of pathogenesis.

## 5. Meta-Inflammation: Alterations of Metabolism Leading to Thrombus Generation

### 5.1. Obesity, Metabolic Syndrome and Dysregulated Lipid Metabolism

Obesity, metabolic syndrome and type 2 diabetes mellitus (T2DM) are among the best-known risk factors for both thrombosis and severe Covid-19 infection since they promote a state of chronic inflammation and impaired fibrinolysis. Meta-inflammation, i.e., chronic inflammation driven by chronically altered metabolic pathways presents with increased release of cytokines from adipocytes. Consequently, a contribution comes from macrophages (recruited in inflamed sites) and transient hypoxia which is proper of visceral fat rather than subcutaneous one.

Macrophages in particular, extend the secretion of cytokines ensuring the vascular endothelium is stimulated towards a pro-thrombotic state with upregulation of adhesion molecules and downregulation of coagulation inhibitory proteins.

Consequently, thrombin generation is increased, platelets are further activated and TNF-α and IL-6 increase the expression of tissue factor, promoting the hypercoagulable state.

Chronic inflammation is also associated with dysregulation of endogenous anticoagulant mechanisms, including tissue factor pathway inhibitor, antithrombin, and the protein C anticoagulation system.

The second important mechanism is impaired fibrinolysis. Expression of PAI-1 is markedly upregulated in visceral adipose tissue and elevated levels of PAI-1, together with tissue plasminogen activator (tPA), have been found in severe Covid-19 patients [121]. 118 hospitalized patients and 30 healthy controls were evaluated through laboratory, imaging, and clot-lysis assays. Elevated levels of those markers were associated with worse respiratory status, enhancement of ex-vivo clot lysis, and mortality. Also, strong correlations were demonstrated with neutrophil counts and circulating calprotectin, a neutrophil activation marker. Plasma levels of PAI-1 are also elevated in patients with obesity or metabolic syndrome. TNF-α is one of the key regulators of PAI-1 expression since it acts by stimulating its expression [122]. This mechanism suggests a clear link between antifibrinolytic activity and obesity-associated chronic inflammatory state.

Recent meta-analyses and case-control studies underlined the association between obesity and severe COVID-19. Also, they showed that obese patients had worse prognoses than non-obese patients (OR, 2.31; 95% CI, 1.3–4.12) [123]. In general, dyslipidemias were also associated with severe COVID-19 (relative risk (RR), 1.39; 95% CI, 1.03–1.87; *p* = 0.03) [124].

### 5.2. Hyperinsulinemia Contributes to Both Impaired Fibrinolysis and Hypercoagulable States

As a causative factor, hyperinsulinemia is related to all the metabolic conditions associated with poor outcomes from Covid-19 infection. Again, the primary mechanism is impaired fibrinolysis, due to increased PAI-1 expression [125]. It, therefore, produces a decrease in plasminogen activator activity.

**Figure 3:**
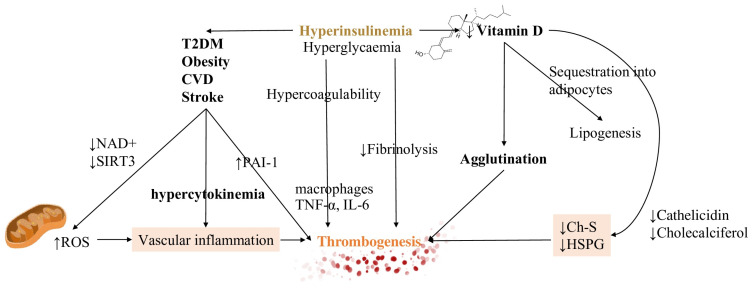
Hyperinsulinemia, CVD and vitamin D have a strong impact on homeostatic equilibrium. Both hyperinsulinemia (depicted in bold and colored font, in order to emphasize it) and hyperglycemia generate states of increased coagulation and decreased fibrinolysis. By driving the development of CVD, diabetes mellitus and obesity, they contribute to the inflammatory substrate of cytokines. They increase the ROS production due to the damage in decreasing both NAD+ and reduced glutathione (GSH). A reduction in vitamin D, due to sequestration into the adipocytes, leads to decreased levels of ChS and HSPG, regulators of RBCs deformation, increasing cells agglutination. These mechanisms are all responsible for thrombosis initiation. Vascular inflammation and decreasing levels of Ch-S and HSPG are represented with colored backgrounds being the main actors of thrombogenesis trigger. Also, thrombogenesis, the main effect, is outlined with a different color too. *Abbreviations*: Ch-S: cholesterol sulfate, HSPG: heparan sulfate proteoglycans, NAD+: nicotinamide adenine dinucleotide, PAI-1 plasminogen activator inhibitor type 1, ROS: reactive oxygen species, T2DM: type 2 diabetes mellitus, SIRT3: sirtuin 3, TNF-α: tumor necrosis factor-α, IL-6: interleukin 6.

The impairment of fibrinolysis by insulin is completely independent of glycemia while hyperglycemia produces effects on coagulation which is not related to insulinemia. Impaired fibrinolysis is consistently found in T2DM patients [126] (Figure 3).

Hyperglycemia instead stimulates clotting factors synthesis from the liver and inflammatory reaction by increasing the secretion of IL-6. Biochemical effects of hyperinsulinemia are several and entail β-oxidation and ketolysis inhibition [127].

Due to the major depletion of nicotinamide adenine nucleotides (NAD+) by glucose oxidation rather than beta-oxidation and ketolysis, NAD+ availability for mitochondrial deacetylase sirtuin 3 (SIRT3) activity decreases [121]. This process, which is NAD+ dependent, normally increases the production of NADPH reduce oxidised glutathione (GSSG) to reduced glutathione (GSH) [128]. Therefore, the final result is that insulin increases mitochondrial production of reactive oxygen species (ROS), also via generation of ceramides [129]. ROS production is then responsible for further vascular inflammation. (Figure 2).

### 5.3. Vitamin D Metabolism and Its Interference in the Inflammatory Pathway

Vitamin D hydroxylation requires magnesium which is notably depleted in hyperinsulinemic conditions. Other mechanisms responsible for decreased Vitamin D levels are increased renal excretion, reduced intracellular levels, and sequestration into adipocytes. The last process is related to lipogenesis which is already increased in hyperinsulinemic patients [128]. Consequences of reduced activation are decreased levels of cholesterol sulfate (Ch-S), heparan sulfate proteoglycans (HSPG), and cathelicidin synthesis. The final result is the promotion of agglutination and, subsequently, of thrombosis. An explanation for this can be found in the action of HSPGs as potent anticoagulant molecules which also buffer glycation damage. Ch-S is instead implicated in RBCs shape deformation for traveling through vascular spaces of reduced caliber [128,129,130].

### 5.4. Extrahepatic Vitamin K Insufficiency and Dependency of Coagulation Factors

Evidence of altered laboratory values of vitamin K have been demonstrated in severe Covid-19 cases. Other than coagulation factors, several other molecules, among which matrix Gla protein (MGP), a potent inhibitor of soft tissue calcification and elastic fibers degradation, are dependent on vitamin K.

The proposed mechanism by Janssen et al. [131], implicates that following elastic fibers degradation due to SARS-CoV-2 proteolysis, partially degraded elastic fibers show increased polarity which drives an increase in the calcium content of elastic fibers.

With an increase in MGP synthesis, vitamin K is utilized for further molecule processing (MGP carboxylation) which decreases circulating vitamin K levels and increases damage to lung tissues. As a consequence, endothelial protein S is not sufficiently carboxylated and shifts the coagulation equilibrium towards thrombogenesis [132].

An important proof that this mechanism may be deranged in Covid-19 thromboembolic events, is that diabetes, hypertension, and CVD, which are correlated with worse outcomes, are conditions related to chronic elastic fibers pathology.

### 5.5. Hormonal Factors Contributing to Covid-19 Thrombotic Complications

Gender differences in Covid-19 outcomes and cardiovascular diseases have raised many issues regarding the hormonal asset of patients and its interference with coagulopathy development.

Endothelial dysfunction is found in the elderly, smokers, and patients with metabolic alterations such as dyslipidemias, T2DM and arterial hypertension [133,134].

An interesting mechanism is the presence of receptors on megakaryocytes and platelet membranes for both estrogen and androgens. Also, testosterone has been observed to increase the secretion of endothelial nitric oxide eNO, a potent inhibitor of platelet activation [135,136].

The function of estrogens, in women, is also directed to regulate and enhance platelet function according to the ovarian cycle release of hormones. On the contrary, lack of testosterone i.e., hypogonadism in the elderly produces a less protective effect on hypercoagulation [137]. Consequently, this aspect should be taken into account when evaluating older patients for possible thrombosis.

## 6. Disorders of Haemostasis and ChAdOx1 nCoV-19 Adenoviral Vector Vaccine

Recently, following the recording of thrombotic adverse events secondary to the administration of the ChAdOx1 nCov-19/AstraZeneca, interest has emerged in evaluating the contribution of antiphospholipid antibodies. A previous study of Zhang et al. [136] reported three cases with severe COVID-19 and cerebral infarction. One of these was associated with bilateral limb ischemia in which a high level of antiphospholipid antibodies was noted.

In March several cases of unexpected thrombotic events and thrombocytopenia that have developed after vaccination with the recombinant adenoviral vector encoding the spike protein antigen of SARS Cov 2 (ChAdOx1 nCov-19, AstraZeneca) were brought to the attention of researchers [137,138,139,140], international drug control authorities, and the WHO [141,142,143,144] (Table 2).

Pathophysiological processes linking the inflammatory response from SARS Cov2 and changes in hemostasis remain unclear. They represent an important step both to prevent the most serious thrombotic complications of Covid-19 disease and to resolve the side effects associated with the administration of the ChAdOx1 nCov-19, AstraZeneca. Besides, whether antiphospholipid antibodies play an important role in either the pathophysiology of COVID-19-associated thrombosis or the hemostatic protuberant effects related to vaccine administration requires further investigation [130]. Several cases have been reported of patients who presented with venous thrombosis and thrombocytopenia 7 to 10 days after administration of the first dose of the ChAdOx1 nCoV-19 adenoviral vector vaccine against coronavirus disease 2019 (Covid-19) (Table 3).

The effectiveness and safety associated with the use of vaccines to combat the 2019 coronavirus pandemic (Covid-19) have been established in several randomized blinded clinical trials [145,146,147,148,149,150].

From December 2020 to March 2021, the European Medicines Agency has established suitability for administration for four vaccines: two messenger RNA-based vaccines—BNT162b2 (Pfizer-BioNTech) and mRNA-1273 (Moderna)—which encode the spike SARS-CoV-2 glycoprotein antigen, encapsulated in lipid nanoparticles; ChAdOx1 nCov-19 (AstraZeneca), a recombinant chimpanzee adenoviral vector encoding the SARS-CoV-2 spike glycoprotein; and Ad26.COV2.S (Johnson & Johnson/Janssen), a type 26 recombinant adenovirus vector encoding the SARS-CoV-2 spike glycoprotein. At the beginning of March 4, the EMA initiated an ongoing review of COVID-19 vaccines produced by Novavax, CureVac AG, and Sputnik V [150]. To date, over 600 million doses have been administered globally [151] while the number of doses that have been administered in the European Union as of 7 April 2021 was more than 82 million doses of the vaccine [152].

Beginning in late February 2021 several cases have been reported of patients who presented with venous thrombosis and thrombocytopenia 7 to 10 days after administration of the first dose of the ChAdOx1 nCoV-19 adenoviral vector vaccine against coronavirus disease 2019 (Covid-19) [137,138].

The immunogenicity benefits associated with the use of ChAdOx1 nCoV-19 have been established in a landmark paper from the Laboratory of Virology, National Institute of Allergy and Infectious Diseases (Hamilton, MT, USA), and from the Jenner Institute, University of Oxford (Oxford, UK) and in an animal study on rhesus macaques [153,154]. The best result using vaccination with ChAdOx1 nCoV-19, with a prime-only or prime-boost regimen, is almost certainly due to eliciting a robust humoral and cell-mediated response. The study reported a response that was predominantly mediated by type 1 helper T cells, as demonstrated by the IgG subclass profile and the expression of cytokines. Probably its peculiar characteristics are explained by the marked tendency to develop significantly lower viral load in the bronchoalveolar lavage fluid and in the tissue of the lower respiratory tract of vaccinated rhesus macaques that have been treated with SARS-CoV-2 compared to control animals. In addition, pneumonia was not observed in animals vaccinated against the SARS-CoV-2 infection. Furthermore, compared to control animals, all animals infected with SARS Cov2 and vaccinated did not develop the disease. The first published single-blind RCT [147] compared results between the vector adenovirus chimpanzee vaccine (ChAdOx1 nCoV-19) expressing the SARS-CoV-2 spike protein and the meningococcal conjugate vaccine (MenACWY). The ChAdOx1 nCoV-19 recruited 543 patients from 5 centers. The population was represented by healthy adults aged 18–55 years with no history of laboratory-confirmed SARS-CoV-2 infection or of COVID-19-like symptoms. The co-primary outcome was to evaluate the vaccine efficacy estimated by the number of confirmed virologically symptomatic COVID-19 cases and the vaccine safety measured by the occurrence of serious adverse events. Secondary outcomes included the safety, reactogenic, and immunogenicity profiles of ChAdOx1 nCoV-19 and efficacy against hospitalized COVID-19 patients. There were no serious adverse events related to ChAdOx1 nCoV-19. Local and systemic reactions were more common in the ChAdOx1 nCoV-19 recipients and included pain, feeling feverish, chills, muscle ache, headache, and malaise (all *p* < 0.05). Many of these reactions were reduced by the administration of prophylactic paracetamol. The immune response was effective in the ChAdOx1 nCoV-19 population, with excellent spike protein-specific T cell response peaking on day 14. Antibody response mediated by anti-spike IgG production was highest by day 28 and was boosted after a second dose. It is important to emphasize that after a booster dose, all participants had a neutralizing activity. Neutralizing antibody responses were strongly correlated with antibody levels measured by ELISA.

While the RCT results on the safety and efficacy of the BNT162b2 mRNA Covid-19 vaccine were awaited [138], the Phase 3 study for the ChAdOx1 nCoV-19 vaccine was initiated to obtain a substantial framework of detailed evidence to support vaccine efficacy and safety for the elderly (age ≥ 70 years), as this population of individuals is at increased risk of serious illness and death if they develop COVID-19 [147].

In a short time, three independent studies supported this finding, not only in larger patient cohorts but also to show that the vaccine would immunize a large portion of the population. A single-blind, randomized study [147] was designed to include healthy adults aged 18 years and older in an age-escalation manner, into 18–55 years, 56–69 years, and 70 years and older immunogenicity subgroup. The study recruited 560 patients (ChAdOx1 nCoV-19 *n* = 300 and MenACWY *n* = 260) and reported 13 serious adverse events that occurred during the study period, none of which were considered to be related to either study vaccine. Regarding local and systemic reactions, they were more common in recipients of the ChAdOx1 nCoV-19 vaccine than in those treated with the control vaccine. The nature of the reactions was similar to that reported in the phase 2 study (injection site pain, feeling of fever, body aches, headache). Side effects were less common in older adults (age ≥ 56 years) than young adults. In recipients of two vaccine doses, median SARS-CoV-2 IgG anti-peak responses 28 days after the boost dose were similar in the three age cohorts. No differences in neutralizing antibody titers were reported after a boost dose which were similar across all age groups [147]. The important immunologically finding was the recording, within 14 days for 99% of the participants receiving the boosted dose, of robust immune responses with neutralizing antibody production. Cell-mediated immune response was also optimal with T lymphocytes peaking on day 14 following the standard single dose of ChAdOx1 nCoV-19. ChAdOx1 nCoV-19 appears to be a safe and effective vaccine and although it appears to be better tolerated in older adults than in younger adults; however, it has similar effect on immunogenicity in all age groups after a boost dose [147]. We summarize in Table 4 major phase 1, 2 or 3 trials and interim analyses reported above.

The second RCT [147] evaluated the safety and efficacy of the ChAdOx1 nCoV-19 vaccine against severe acute respiratory syndrome coronavirus 2 (SARS-CoV-2), in a pooled interim analysis of four RCTs that included a large population of 23,848 individuals. Participants were enrolled and 11 636 participants (7548 in the UK, 4088 in Brazil) were included in the interim primary efficacy analysis. In recipients two standard doses of ChAdOx1 nCoV-19 vaccine (*n* = 4440) efficacy was 62.1% (95% CI 41.0–75.7; 27 [0.6%] compared to 71% in control population. Enrolled individuals were 18 years of age or older and were randomly assigned (1:1) to ChAdOx1 nCoV-19 vaccine or control (meningococcal group A, C, W, and Y conjugate vaccine or saline). Recipients who were managed with a low dose followed by a standard dose showed efficacy that was 90.0% (67.4–97.0; three [0.2%] of 1367 vs. 30 [2.2%] of 1374; *p* interaction = 0.010). There were ten hospitalized cases for COVID-19 after the 21st day of the first dose and all were included in the control population. Of these two were classified as severe COVID-19, one died. There were 74,341 person-months of safety follow-up (median 3–4 months, IQR 1···3–4···8) with a total of 175 serious adverse events recorded in 168 participants. Of these 84 events occurred in the ChAdOx1 nCoV-19 group and 91 in the control group. Of note, three events were classified as possibly related to a vaccine: one in the ChAdOx1 nCoV-19 population, one in the control population, and one in a participant who remained masked by the group allocation [147].

The third prospective cohort study [156] compared the effectiveness of the first dose of BNT162b2 mRNA (Pfizer-BioNTech, Magonza, Germany) and ChAdOx1 (Oxford-AstraZeneca, Cambridge, UK) in preventing hospital admissions. The authors demonstrated that recipients of the first dose of the BNT162b2 vaccine showed an effect of 85% (95% confidence interval [CI] 76 to 91) for COVID-19-related hospitalization at 28–34 days after administration of the vaccine. Contrary to an analysis of the effect of the ChAdOx1 vaccine over the same time interval it was noted that this had an effect of 94% (95% CI 73–99) in protecting against hospitalization. The most interesting data of this study concerns the analysis in the subpopulation of older individuals. In fact, the results of the combined effect of the vaccine for the prevention of hospitalization related to COVID-19 were comparable by narrowing the analysis to subjects aged ≥ 80 years (81%; 95% CI 65 to 90 at 28–34 days after vaccination). The limitation of this study was that no serious side effects were shown.

## 7. Future Directions and Perspectives

Despite the efforts research has made in the past year to formulate optimal therapies for the treatment of SARS-CoV-2 infection, the results have been disappointing in both patients with mild COVID-19 and those hospitalized in the critical phase of the disease. Two very recent RCTs depict a frustrating scenario.

The use of ivermectin for treatment was evaluated in a very recent RCT [157]. Mildly symptomatic Covid-19 patients receiving 5 days of ivermectin therapy compared to placebo population did not experience significant improvements in symptom resolution time. Although the results of using ivermectin have not shown a treatment benefit in recipients with mild Covid-19, larger studies may be needed to understand the effects of ivermectin on candidates with more severe forms of the disease to evaluate whether the drug has clinically relevant effects [156].

Thrombotic events are commonly reported in critically ill patients with Covid-19. As previously illustrated, there is limited data to guide antithrombotic prophylaxis. In the INSPIRATION investigation trial [158], authors studied patients for a 30-day follow-up. One population of patients received intermediate-dose (enoxaparin, 1 mg/kg daily) (*n* = 276) and was compared to those who were managed with standard prophylactic anticoagulation (enoxaparin, 40 mg daily) (*n* = 286). The results did not record a significant difference considering the primary outcome set on composite of venous or arterial thrombosis, on treatment with extracorporeal membrane oxygenation, or mortality within 30 days among patients admitted to intensive care with COVID-19 between the groups. Regarding unselected patients admitted to intensive care with COVID-19, the evidence does not support the routine empirical use of prophylactic anticoagulation at intermediate doses [158].

Another concern has been for many critically ill hospitalized patients who required immediate surgery [151,152,155]. and in which Sars-Cov-2 was not even considered at presentation. Therefore, a vacancy was created in the predefined guidelines raised doubts about the timing in which patients acquired the COVID-19 disease, fueling the suspicion that the infection had occurred in the preoperative period which was therefore not negligible [159,160,161].

Mass vaccination however raises the possibility of preventing death and avoiding hospitalization in critical conditions. In this regard, data from Israel, which is currently leading the world in terms of percentage of the vaccinated population, are comforting [162]. Covid-19 cases and hospitalizations recorded a significant decrease in mid-January. The greatest effects were related to the massive administration of the vaccine among older individuals, who were prioritized for vaccination. The Israeli health authority reported a reduction in Covid-19 hospitalizations by 36% and 29% with a significant decrease in patients experiencing clinically more severe Covid-19 compared to 3 weeks earlier. The main concern is variants of the original Wuhan-Hu-1 spike protein. Variant B.1.1.7, first identified in the UK, is now the dominant genetic variant of SARS-CoV-2 in both Israel and the UK. This variant does not appear to reduce neutralizing antibodies to the same extent as the South African variant B.1.351. We must be persuaded only the real-world experience can currently provide answers to the efficacy of Covid-19 vaccines against SARS-CoV-2 disease and death as well as its variants [163]. International cooperation has acquired a fundamental role in sharing and distributing large-scale data through verification of official sources (Table 5).

## 8. Conclusions

The systemic complications of the COVID-19 infection is due to a combination of factors as elaborated above. Treatment should be tailored to the patient with an escalating plan dictated by clinical severity. There are several mechanisms to explain the potential thrombotic complications by both the COVID-19 infection and the vaccines which should be considered. Despite the thrombotic complications, vaccines have been shown to reduce hospital admissions and the severity of illness in robust studies. Long term effects of these however remain uncertain.

## Figures and Tables

**Table 1:** 2020 case-control retrospective studies comparing risk factors for thrombosis development in hospitalized patients with severe Covid-19 (controls) versus hospitalized patients with both severe infection and DVT or ATE (cases). VTE: venous thromboembolism, ATE: arterial thromboembolism, WBCs: white blood cells, INR: international normalized ratio, aPTT: activated partial thromboplastin time, CRP: C reactive protein, ICU: intensive care unit, CTPA: CT pulmonary angiography, IL-6: interleukin-6, DVT: deep vein thrombosis, IMV: invasive mechanical ventilation.

Reference	Total SARS-CoV-2 + Hospitalized Patients	VTE and ATE Cases	Risk Factors More Present in Cases (*p* < 0.05)	Risk Factors Similar in Cases and Controls (*p* > 0.05)	Conclusions
Stoneham et al., 2020 [26]	208	21	High Wbcs, high D-dimer, high INR.	aPTT ratio, fibrinogen.	Comorbidities were not associated with a higher risk of thrombosis. Monitoring of D-dimer and anti-factor Xa levels may be relevant for management.
Zuo et al., 2020 [27]	44	11	High calprotectin, markers of NETs (myeloperoxidase-DNA complexes) high D-dimer, high platelets.	Troponins, Wbcs.	There was a significant difference between peak D-dimer, calprotectin and cell free DNA levels between the populations.
Zhang et al., 2020 [28]	143	66	High Wbcs, older age, low oxygenation index, high rate of cardiac injury, CURB-65 score 3 to 5, Padua score ≥ 4, high D-dimer.	Platelets count.	COVID-19 is suspected to cause an additional risk factor for DVT in hospitalized patients.
Planquette et al., 2020 [29]	1042	59	High CRP, fibrinogen, D-dimer. IMV.	Comorbidities: BMI, previous VTE, ATE, Cancer, hypertension, Cardiovascular diseases.	Similar prevalence of VTE risk factors in cases and controls was found. In both groups, altered coagulation parameters were found.
Trimaille et al., 2020 [30]	289	49	High Improve score, high WBCs, D-dimer, low haemoglobin at discharge.	Padua score of 4 or more, CRP	Lack of thromboprophylaxis is a major determinant of VTE in non-ICU COVID-19 patients. Comorbidities were not found to affect the event occurrence.
Shah et al., 2020 [31]	187	81	High troponins, ferritin, D-dimer.	Platelets count, Wbcs, thromboelastography parameters.	Elevated D-dimer, ferritin, troponin and white cell count at ICU admission may reflect undiagnosed altered coagulation and be used to identify patients for CTPA.
Kolielat et al., 2021 [32]	117	18	High D-dimer, fibrinogen, ferritin.	Wbcs, platelets, troponins, Il-6.	Elevated D-dimer and a less elevated fibrinogen are associated with DVT despite conventional thromboprophylactic treatment.
Kampouri et al., 2020 [33]	443	41	High D-dimer, positive Wells criteria, bilateral infiltrates on X-rays or CT scan, mechanical ventilation.	Wbcs, platelets, CRP, Padua score, Geneva score.	The combination of Wells ≥ 2 score and D—dimer ≥ 3000 ng/L is predictive of VTE at admission. Hospitalization in the ICU and especially mechanical ventilation were associated with VTE occurrence. The combination of Wells’ score and D-dimer value can be used for guiding empiric anticoagulation therapy.

**Table 2:** Investigational studies related to ChAdOx1 nCov-19-associated thrombosis cases. We reported major findings, age (as median or mean) and female gender prevalence in number &/or percentage. PF: platelet factor.

Published Study	Vaccine Type	Patient (N)	Women (N)	Age	Time Span	Cases’ Etiology	Major Findings
Greinacher et al. [137]	ChAdOx1 nCov-19	11	9	36 yrs (median)	5 to 16 days after 1st dose	10 multiple thrombosis, 9 cerebral venous thrombosis. 3 splanchnic-vein thrombosis. 3 pulmonary embolisms, and 4 others. 5 disseminated intravascular coagulation	Immune thrombotic thrombocytopenia. High level of Platelet-activating antibodies against PF4 mimicking autoimmune heparin-induced thrombocytopenia.
Schultz et al. [138]	ChAdOx1 nCov-19	5	4/5	40.8 ys (mean)	7 to 10 days after	2 thromboses (sigmoid cerebral sinuses), 1 thrombosis (portal vein branches) 1 massive thrombosis plus right cerebellar hemorrhagic infarction 1 massive cerebral vein thrombosis with global edema.	High levels of antibodies to platelet factor (PF) 4-polyanion complexes. Authors propose the acronym VITT (vaccine-induced immune thrombotic thrombocytopenia) as causative mechanism.

**Table 3:** Recent rare cases’ reports of thrombosis following administration of anti-SARS-CoV-2 vaccines. Data for ChAdOx1 nCov-19 vaccine are reported by 4 April 2021. Data for JNJ-78436735/Ad26.COV2.S are reported by 26 April 2021. CVST: cerebral venous sinus thrombosis, EEA: European Economic Area, UK: United Kingdom, US: United States.

Vaccine Type	Thrombosis Cases	Total Administrations	Associated Factors
ChAdOx1 nCov-19	169 CVST, 53 splanchnic vein thrombosis	34 million people had been vaccinated in the EEA and UK	<60 years of age, symptoms onset within 3 weeks after vaccination, female gender, thrombocytopenia.
JNJ-78436735/Ad26. COV2. S	6 CVST (1 death)	8.09 million in US	Thrombocytopenia, women between 18 and 48 ys. symptoms onset between 6 to 13 days after vaccination

**Table 4:** Selected studies investigating efficacy and adverse events of most administered anti-SARS-CoV-2 vaccines worldwide. For each study, the total participants number, women/total number and age are presented as baseline characteristics. Major findings have been divided into two columns: adverse events and efficacy data in terms of prevention of Covid-19 and antibodies titers. Data are number (percentages).

Published Study	Vaccine Type	Participant (*n*)	Women (*n*)	Age	Vaccine Components	Adverse Events	Efficacy
Voysey et al. (interim analysis of COV001, COV002, COV004, COV005) [147]	ChAdOx1 nCoV-19	11,636 5807-va 5829-ca	3525/5807	mostly 18–55 yrs	dsDNA encoding for the Spike protein protected in an adenoviral particle	175 severe adverse events	2 standard doses efficacy was 62.1%. Low boosted dose efficacy was 90.0%.
Ramasamy et al. (phase 2 of COV002) [148]	ChAdOx1 nCoV-19	560 420-va 140-ca	104 low-d 101 standard-d	100 (18–55 yrs) 120 (56–69 yrs) 200 > 70 yrs	dsDNA encoding for the Spike protein protected in an adenovirus.	Systemic reactions 86% (18–55 yrs) 77% (56–69 yrs) 65% >70 yrs	14 days after the 2nd dose, 208 of 209 boosted participants had neutralising antibody responses. T-cell responses peaked at day 14 after a single dose.
Logunov et al. (phase 3) [150]	rAd26 and rAd5 vector-based vaccine (Sputnik V)	21.977	5821 (38.9%)	mostly 18–60 yrs	dsDNA encoding for the Spike protein protected in Ad26 vector for the 1st dose, Ad5 for the 2nd one).	4 deaths, none was related to the vaccine.	Vaccine efficacy was 91.6% (95% CI 85.6–95.2).
Zhang et al. (phase 1/2) [155]	CoronaVac (Sinovac Life Sciences, Beijing, China)	743	397/743	phase 1 42.6 yrs phase 2 42.1 yrs	Inactivated virus vaccine with beta-propiolactone	In the phase 2 trial adverse reactions was 19% with 3 μg 19% with 6 μg group, and 18% with placebo.	In the phase 2.97% seroconversion with 3 μg, 100% with 6 μg group, and 0% with placebo group.
Sadoff et al. (interim analysis of phase 1–2) [149]	Ad26.COV2. S/JNJ-78436735 (Johnson&Johnson)	805	169 in cohort 1, 159 in cohort 3	35.4 ± 10.2 (cohort 1) 69.8 ± 4.0 (cohort 3)	dsDNA encoding for the Spike protein protected in an adenoviral particle (Ad26)	The most frequent systemic adverse event was fever. Systemic adverse events were less common in cohort 3 than in cohort 1.	Reactogenicity was lower after the second dose. Neutralizing-antibody titers were detected in 90% or more of all participants on day 29 after the first vaccine dose
Polack et al. [145]	BNT162b2 mRNA	43.548	9221 (48.9)	52.0	mRNA vaccine encoding for Spike protein protected in a lipid nanoparticle	Serious adverse events incidence was low and similar between the vaccine and placebo groups.	BNT162b2 95% effective in preventing the disease. Similar efficacy for age, sex, race, ethnicity, baseline body-mass index, and the presence of coexisting conditions.
Baden et al. [146]	mRNA-1273 vaccine (Moderna)	30.351	7108 (46.9)	51.3 (18–95)	encapsulated mRNA vaccine encoding for Spike protein protected in a lipid nanoparticle	Transient local systemic reactions. No safety concerns were identified.	mRNA-1273 94.1% effective in preventing severe and mild disease development.

Abbreviation. Ca = control administration; dsDNA = double strand DNA, va: vaccine administration.

**Table 5:** Total administrations of anti-SARS-CoV-2 vaccines according to selected countries and type of vaccine. Data are only available for countries which provided official reports. Time span is 24 December 2020–26 April 2021.

-	Adenoviral Vector				mRNA	
Country	ChAdOx1 nCov-19 (Astrazeneca)	Sputnik V/Gam-Covid-Vac	CoronaVac (SinoVac)	JNJ-78436735/Ad26.COV2.S (Johnson&Johnson)	BNT162b2 mRNA (Pfizer)	mRNA-1273 (Moderna)
France	3.72 million	0	0	2004	14.33 million	1.59 million
Germany	5.60 million	0	0	0	18.81 million	1.47 million
Italy	3.97 million	0	0	20700	12.83 million	1.27 million
United States	0	0	0	8.09 million	116.19 million	100.83 million

Source of data: https://ourworldindata.org/covid-vaccinations (accessed on 10 April 2021); mRNA-1273 Moderna: https://github.com/owid/covid19data/tree/master/public/data/vaccinations/locations.csv (accessed on 10 April 2021); ChAdOx1 nCoV-19: https://github.com/owid/covid-19-data/tree/master/public/data/vaccinations/locations.csv (accessed on 10 April 2021); BNT162b2 mRNA: https://github.com/owid/covid-19-data/tree/master/public/data/vaccinations/locations.csv (accessed on 10 April 2021); CoronaVac (SinoVac): https://github.com/owid/covid-19-data/tree/master/public/data/vaccinations/locations.csv (accessed on 10 April 2021); JNJ-78436735/Ad26.COV2.S (Johnson & Johnson): https://github.com/owid/covid-19-data/tree/master/public/data/vaccinations/locations.csv (accessed on 10 April 2021).

## Abbreviations

TLR7: Toll-like receptor 7, MI: Myocardial infarction, VTE: venous thromboembolism, FDA: Food and Drug Administration, UFH: Unfractionated Heparin, LMWH: low molecular weight heparin, DOAC: direct oral anticoagulants, aPTT: activated partial thromboplastin time, INR: international normalized ratio, PE: pulmonary embolism, ECMO: extracorporeal membrane oxygenation, PT: prothrombin time, ACS: acute coronary syndromes, PCI: percutaneous coronary intervention, SIRS: systemic inflammatory response syndrome, HIV: human immunodeficiency virus, PDGF: platelet-derived growth factor, MHC: major histocompatibility complex, CD: cluster of differentiation, CTLA: cytotoxic T-lymphocyte antigen, PD: programmed cell death protein, PD-L: programmed cell death ligand, TNF-α: tumor necrosis factor-α, PAI-1: plasminogen activator inhibitor-1, OR: odds ratio, CI: confidence interval, CVD: cardiovascular disease, eNO: endothelial nitric oxide, EMA: European Medicines Agency, ELISA: enzyme-linked immunosorbent assay, RCT: randomized controlled trial.
